# Surgical Medical Education via 3D Bioprinting: Modular System for Endovascular Training

**DOI:** 10.3390/bioengineering11020197

**Published:** 2024-02-19

**Authors:** Ruben Foresti, Anna Fornasari, Claudio Bianchini Massoni, Arianna Mersanne, Chiara Martini, Elisa Cabrini, Antonio Freyrie, Paolo Perini

**Affiliations:** 1 Department of Medicine and Surgery, University of Parma, Via Gramsci 14, 43126 Parma, Italyantonio.freyrie@unipr.it (A.F.); 2 Center of Excellence for Toxicological Research (CERT), University of Parma, 43126 Parma, Italy; 3 Italian National Research Council, Institute of Materials for Electronics and Magnetism (CNR-IMEM), 43124 Parma, Italy; 4 Vascular Surgery, Cardio-Thoracic and Vascular Department, University-Hospital of Parma, 43126 Parma, Italy; anna.fornasari91@gmail.com (A.F.); claudiobianchinim@gmail.com (C.B.M.); arianna.mersanne@ao.pr.it (A.M.); elisacabrin@gmail.com (E.C.); 5 Diagnostic Department, University-Hospital of Parma, Via Gramsci 14, 43126 Parma, Italy

**Keywords:** 3D printing, 3D model, surgical education, surgical simulation, endovascular surgery, vascular surgery

## Abstract

There is currently a shift in surgical training from traditional methods to simulation-based approaches, recognizing the necessity of more effective and controlled learning environments. This study introduces a completely new 3D-printed modular system for endovascular surgery training (M-SET), developed to allow various difficulty levels. Its design was based on computed tomography angiographies from real patient data with femoro-popliteal lesions. The study aimed to explore the integration of simulation training via a 3D model into the surgical training curriculum and its effect on their performance. Our preliminary study included 12 volunteer trainees randomized 1:1 into the standard simulation (SS) group (3 stepwise difficulty training sessions) and the random simulation (RS) group (random difficulty of the M-SET). A senior surgeon evaluated and timed the final training session. Feedback reports were assessed through the Student Satisfaction and Self-Confidence in Learning Scale. The SS group completed the training sessions in about half time (23.13 ± 9.2 min vs. 44.6 ± 12.8 min). Trainees expressed high satisfaction with the training program supported by the M-SET. Our 3D-printed modular training model meets the current need for new endovascular training approaches, offering a customizable, accessible, and effective simulation-based educational program with the aim of reducing the time required to reach a high level of practical skills.

## 1. Introduction

Surgical skill training is moving away from the Halstedian principle of “learning by doing” and establishing simulation as a pivotal tool to provide trainees with the necessary skills and competencies [1]. In fact, traditional surgical training during real-life interventions in the operating room (OR) has been reported to be inefficient, endangering patient safety and prolonging procedure time [2]. Even though the importance of training based on simulations is well documented [3], the use of simulators is still limited, mainly due to their very high cost. Therefore, to reduce healthcare costs through improved OR efficiency and reduction of medical errors [4,5], it is strongly recommended to include simulation-based training outside the OR. A particular attention to cost-effectiveness is crucial to making simulations a routine part of endovascular specialists’ training programs.

Three-dimensional (3D) printing is a growing technology that is changing the manufacturing industry and offers many advantages over traditional manufacturing, including the ability to create objects with complex internal structures, improved versatility, customization, and lower space requirements [6,7], reducing costs related to injection molding, and low production numbers (typically one patient requires one custom device). 3D-printing may offer, at the same time, a highly immersive and tactile learning experience. Thanks to the possibility to print multiple materials in the same fabrication procedure, bioprinting supports tissue regeneration via synthetic biology and surgery training via bionic models.

The purpose of this study was to develop and evaluate a 3D-printed modular model for endovascular training (M-SET), with the aim of providing a new educational tool for endovascular trainees to develop the tactile sensation of handling a guidewire while crossing atheromatous plaques. The 3D-printed model was developed to simulate an endoluminal treatment of a femoro-popliteal segment via an antegrade femoral puncture. The research question focused on assessing the model’s Impact on trainees’ learning experiences through questionnaires and evaluations by senior surgeons.

### 1.1. Simulation and Training in Vascular Surgery

Over the last decades, vascular surgery has been transformed by technological advancements, shifting away from open surgical vascular operations to minimally invasive endovascular treatment (EVT). In fact, EVT is now considered the first-choice treatment for many vascular diseases needing intervention. Concurrent with these trends, the vascular surgery trainee experience has changed to reflect ongoing demands on the specialty and surgical training. While conflicting evidence in the literature makes it difficult to interpret the full impact of these changes, there is concern among surgical educators and residents that graduating trainees may be unprepared to independently practice the full spectrum of vascular surgery [8,9,10].

In detail, the traditional teaching approach of “see one, do one, teach one” is undergoing a transformation, with a contemporary emphasis on “see one, sim many, do one” [11]. Surgical skill and simulation centers have been created at many centers in Northern America and Europe. Simulation training has been adopted by medical educators to accelerate psychomotor skills acquisition, enhance the learning curve of new skills, and improve procedural understanding [12,13,14,15]. In fact, simulation allows repeated practice, and trainees may learn from their mistakes away from real patients [16], defining techniques to replace real patient experiences with artificially contrived and guided experiences that replicate substantial aspects of the real world in a fully interactive manner [3]. Endovascular procedures require a high degree of precision and skills that were traditionally taught exclusively through an apprenticeship model based entirely on patients. In the latest meta-analysis conducted by Haiser et al., there is a notable shift towards educational validation and increased utilization of high-fidelity virtual/mixed reality (VR/MR) simulators for endovascular training [17,18]. Among the prominently recognized simulators are ANGIO Mentor (Simbionix, Cleveland, OH, USA), Vascular Intervention System Trainer (VIST) simulator (Mentice AB, Gothenburg, Sweden), and SimSuite (Medical Simulation Corporation, Denver, CO, USA) [19]. The costs of simulators for endovascular surgery can vary widely based on factors such as complexity, features, and realism. Entry-level virtual reality (VR) simulators for endovascular training might cost in the range of 30,000 to 50,000€. These systems typically provide fundamental skill training and basic procedural simulations. More advanced VR simulators with additional features, such as haptic feedback and realistic anatomical models, can range from 50,000 to 100,000€ or more [20]. However, studies in the literature confirm that basic skills trainers, which are accessible to most teaching hospitals and priced similarly to a sophisticated laptop, still demonstrate adequate efficacy and are considered valuable additions to surgical training curricula [21]. Cadaveric simulation is another option for endovascular training, especially to develop tactile skills; however, it is acknowledged to be both expensive and often impractical [22].

Finally, both VR/MR and cadaveric endovascular simulation are associated with substantial financial costs and the need for ongoing technical support. In response to these challenges, there is a growing interest in developing simpler, low-cost, and maintenance-free endovascular simulators. The aim was to enhance training effectiveness and overcome barriers hindering the broader adoption of simulation training [23,24].

### 1.2. 3D Printing and Bioprinting in the Healthcare Context

3D printing is a set of production processes for additive manufacturing (AM). The material is deposited layer-by-layer, enabling the ability to create objects with complex shapes not attainable with typical material subtraction manufacturing processes. At the same time, 3D printing utilizes only the required material, reducing waste and pollution [25]. Bioprinting identifies all AM processes that aim to combine cells, growth factors, and/or biomaterials to fabricate biomedical parts, frequently to achieve characteristics that mimic natural tissue or its morphology, enabling the rapid customization of personalized devices and/or drugs to support tissue functionality restoration. A biomaterial is a substance (derived either from nature or synthesized in a laboratory) that has been engineered to interact with the biological systems for a medical purpose, either a therapeutic (i.e., treat, augment, repair, or replace a tissue function of the body) or a diagnostic one.

Starting from magnetic resonance imaging or computer tomography, it is possible to achieve a 3D digital anatomical model and fabricate the final device, aiming to support better surgical planning and tissue regeneration [26,27,28]. The first approach is already used in different fields of surgery, such as cardiovascular [29], thoracic [30], facial plastic and reconstructive [31], eye care [32], otolaryngology [33], cranio-maxillofacial [34], cranial neurosurgery [35], spinal [36], and orthopaedic surgery [37] and has demonstrated the potential to reduce errors and costs. Hence, in the healthcare context, it is mandatory to evaluate AM families, select the technologies/materials able to reproduce the required medical device, and meet all of the governance requirements [38]. Moreover, the selected procedures have to confirm the safety, compatibility, timing, and mechanical limitations [39], identify or develop new cost-effective biocompatible and sterilizable materials able to assure accuracy [40,41], precision, and high quality during device development and after post-processing/finishing procedures [42].

The International Organization for Standardization/American Society for Testing and Material (ISO/ASTM) 52900:2021 regulated all the AM technology names and the manufacturing approach into seven families, differentiated by printing head or manipulated material [43]. In detail, sheet lamination or hybrid technology, such as the lift [44,45], is a family and technology based on laminated material. They cut or stimulate special foil by laser or fix and color the standard paper via ink-jet printing head. These fabrication approaches give us the possibility to print multi-material or multi-color devices, but the excess material has to be removed manually, when possible. Powder bed fusion [46], binder jetting [47], and directed energy deposition technologies manufacture the 3D models by fusing or adding chemical agents to the powder, extruded or contained inside a binder [48]. The manufacturing procedures results in a biocompatible or colored object based on the sole material used (is not possible to stratify different materials, without strongly customizing the machine). Therefore, to satisfy the necessity of biocompatible colored materials, we can move to the AM families based on liquid cartridges. Material extrusion approaches can be used to print filaments and fluids, and it is the most flexible AM family, thus used for custom device fabrication (i.e., implantable sensors, biodegradable orthoses), but with a limited fabrication speed [28,49]. Material-jetting is characterized by high resolution and speed via the only deposition of the liquid material from the ink-jet head (based on piezoelectric actuators) and is limited by the viscosity that has to be preserved under 100 cP [50,51]. Finally, renouncing the color to reach the ability to manage any viscosity/performance (i.e., rigidity and flexibility) of the photosensitive resin, VAT polymerization demonstrated to be reliable and cheapest, even though it was limited to the single resin contained in the cartridge [52].

Therefore, towards 5D printing, merging data used to create 3D models with data regarding physiological activity for personalized therapy technique, it is required the development of digital bio-libraries linked to a specific pathology and processing data for disease treatment [30,53,54]. The pathological bio-library must take into consideration all the aspects related to the development of the aforementioned devices, taking into account all the phases required to achieve zero-failure therapeutic continuity and smart bioprinting, starting from the shape and perception design.

## 2. Materials and Methods

### 2.1. Anatomical Accuracy

Measurements from all the preoperative computed tomography (CT) angiography (CTA) scans of patients who underwent, between December 2022 and May 2023, endovascular recanalization of the superficial femoral artery (SFA) in our center were extracted. All CTA were performed in the Radiology Unit of the University Hospital of Parma on the same device, with patients in supine position using 128-slice multidetector CT scanners (SOMATOM Definition Flash, Siemens Healthcare, Forchheim, Germany). All patients underwent a preoperative 3DCT protocol composed of three different scans: un-enhanced CT scanning (SMCT), followed by an angiography, and then a venous phase (CTV) using conventional parameters. The following examination protocol was used: 80–120 kVp (single energy mode) [according to patient body mass index (BMI)], mean X-ray tube current was 240 mAs ref. (automated tube current modulation [CARE Dose 4D was used]), slice thickness/increment 1.00/0.7 mm, kernel Bv40f/I30f (iterative reconstruction, ADMIRE strength 3), single collimation width 0.6 mm, rotation time 0.50 s, matrix 512 × 512. For contrast enhancement, 70–100 mL of contrast medium (Iomeprol, Iomeron 400, Bracco, Milan, Italy) was administered intravenously at a 4–5 mL/s flow rate, followed by 40–50 mL of saline chaser at the same flow rate using an automatic dual-head pump injector (Stellant, MedRAD, Pittsburgh, PA, USA). We used a bolus tracking technique (CARE bolus, Siemens, Forchheim, Germany), and we extended the anatomical coverage from the diaphragm to the feet. 3D post-processing reconstructions were generated by the same researcher (CM) using the imaging software Syngo.via (Siemens Healthcare GmbH, Erlangen, Germany—version VB60A_HF06). The arterial phase was assessed by 2 vascular surgeons (PP and CBM), each with >10 years of experience in the analysis of CTA images with dedicated software and in arterial surgery of the lower limbs, blinded to patients’ clinical data and previous diagnosis. The Digital Imaging and Communications in Medicine (DICOM) files of patients’ CTA images were imported into OsiriX MD 14.0 medical imaging software (Pixmeo SARL, Geneva, Switzerland) and were reconstructed along the central lumen line. Data included: diameter of SFA, total length of SFA, number and length of atheromatous plaques, and minimum lumen diameter measured in the most stenotic region. Means and standard deviations for all registered data were calculated and used as templates for model design and conceptualization.

### 2.2. Design and Conceptualization

The model was created to be simple to use in order to allow trainees to perform simulations autonomously. The main goal of the model was to develop the tactile sensation of navigating with a guidewire in a blood vessel, with different shapes of atheromatous plaques and degrees of stenosis.

The M-SET aimed to reproduce and develop the following tactile sensations:

- Torquability: the response of the tip of the guidewire to the physician’s rotational movement, maintaining control and precision;
- Trackability: the capability to follow the desired path to reach the target site;
- Tactile feedback from the guidewire: increased resistance or friction during guidewire advancement attempts (the guidewire is encountering difficulty in navigating through the vessel or is not following the intended path).

### 2.3. Material Selection and 3D Printing Process

The M-SET was designed with SolidWorks^®^ v. 2015 (Solidsolution, London, UK), converted into .STL files by using Slic3r™ (https://slic3r.org/) (accessed on 21 December 2023), and sent to the printer (including the architectural supports) by using the 3D-printer dedicated software (PreForm 3.28.0, Formlabs, Somerville, MA, USA). All the simulator parts were 3D printed via stereolithography technology (Formlabs2, Formlabs, Somerville, MA, USA) by using photo-responsive medical-grade resin Dental LT V2 (Formlabs, Somerville, MA, USA). After that, the printed objects were washed into isopropyl alcohol (GIP103, Girelli Alcool, Milano, Italy) for 25 min and subsequently cured through UV light installed into the Form Cure (Formlabs, Somerville, MA, USA). Then, the M-SET lid was polished by applying Plastik 70 Kontakt Chemie spray (CRC Industries Europe BVBA, Zele, Belgium) to achieve full transparency and allow trainees to visualize the guidewire’s path and movements while performing the simulation without the need for X-rays or other additional tools.

### 2.4. Evaluation and Testing

#### 2.4.1. Participants

We included in this single-blind randomized study all Vascular Surgery and Interventional Radiology residents in the University Hospital of Parma willing to participate in the study. Demographics (age, gender) and previous experiences, that could be relevant to the residents’ ability to assimilate guidewire’s handling techniques, were acquired by using an entrance survey. The trainees provided and signed informed consent to participate in the study. Twelve trainees were enrolled in the study (4 males and 8 females). Trainees’ ages ranged between 26 and 32 years. Eight out of twelve trainees were part of the Vascular Surgery residency program, while four were part of the Interventional Radiology residency program. All trainees were working in the same teaching hospital at the time of the study. Trainees had different levels of prior experience with endovascular interventions: 10 participants had completed at least 1 year of residency training, and they reported an average of 4 prior peripheral EVT cases as first operators; the other 2 residents reported no prior experience with endovascular procedures. Six residents were randomized into the SS group and performed the training sessions with the model assembled in the simple, medium, and difficult configurations; six residents were randomized into the RS group, which performed the training sessions with the model assembled in two random configurations followed by one difficult configuration. Demographic data of the trainees are summarized in Table 1.

#### 2.4.2. Study Design

The trainees were randomized into two groups, stratified by their postgraduate level (year 1 to year 5); the standard simulation (SS) group performed two training sessions with a standard and stepwise difficulty 3D model, while the random simulation (RS) group performed two training sessions with a random difficulty 3D model. The trainees were blinded regarding their randomization status. The trainees completed a 1 h free, preliminary training session with a random difficulty 3D model to familiarize themselves with the simulation modality. Subsequently, they performed two unsupervised training sessions, according to their randomization group, and one final training session with the highest-level difficulty 3D model, supervised and evaluated by a senior surgeon (vascular surgeon with more than 10 years of experience in peripheral arterial EVT and who performed > 100 endovascular inferior limb revascularization) using the Global Rating Scale of Endovascular Performance [55]. All the training sessions were video-recorded, timed, and considered completed when the guidewire had successfully passed through all the stenoses and the end side of the model.

The trainees, previously instructed on different types of guidewires and catheters, could freely choose the type of guidewire to use if either floppy 0.035 guidewire (Terumo, Shibuya City, Tokyo, Japan) or Sparatcore 0.018 or 0.014 guidewires (Abbott, Chicago, IL, USA), and they had access to additional materials such as 4 Fr BER Tempo Aqua catheter (Cordis, Hialeah, FL, USA), 4 Fr Vertebral Glidecath catheter (Terumo, Shibuya City, Tokyo, Japan), 0.035, 0.018, and 0.014 guidewire torque. At the end of the simulation, trainees provided their experience usefulness evaluations, completing the Student Satisfaction and Self-Confidence in Learning Scale [56].

**Figure 1:**
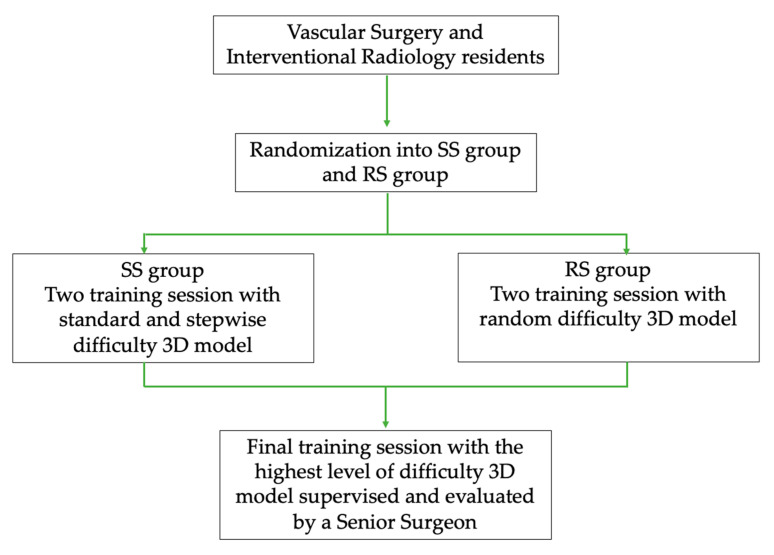
Study design flowchart graph with the phases of the study.

A study flowchart graph with the phases of the study is depicted in Figure 1.

#### 2.4.3. Evaluation Instruments

The Global Rating Scale of Endovascular Performance [55] was developed to assess the essential steps in performing an antegrade SFA angioplasty (excluding the steps required to gain arterial access). A modified Delphi method was used to validate the contents of the rating scale. The internal consistencies (Cronbach α) in the first and second rounds of the Delphi study were 0.89 and 0.856, respectively. The Student Satisfaction and Self-Confidence in Learning Scale [56] is a 13-item scale used to measure student satisfaction with simulation activities (5 items) and self-confidence in learning (8 items). Cronbach α for satisfaction items was 0.94, and for self-confidence, it was 0.87.

### 2.5. Statistical Analysis

The results from the CTA image analysis, demographic data entry survey, the training sessions, and both assessment tools (the Global Rating Scale of Endovascular Performance and the Student Satisfaction and Self-Confidence in Learning Scale) were entered into an .xls spreadsheet (LibreOffice Calc 7.3.7, The Document Foundation, Berlin, Germany) and subsequently analyzed using SPSS 19 (IBM, Armonk, NY, USA). Anatomical accuracy data for the 3D model design was evaluated by mean and standard deviation (SD), or by median and range in the case of a skewed distribution. Training session times and Global Rating Scale of Endovascular Performance data were assessed through the mean ± SD. An unpaired T test was used to test for statistically significant differences. Differences were considered significant with a *p* value of less than 0.05.

## 3. Results

### 3.1. Anatomical Accuracy

Sixteen CTA were obtained and evaluated for the purpose of this study, in order to create a 3D-printed model as close as possible to the real-world atherosclerotic femoral-popliteal lesions we normally treat endovascularly. The mean diameter of SFA was 9.03 ± 1.36 mm, and the mean total length of SFA was 347.56 ± 56.22 mm. The number of plaques varied from 1 to 3 for each vessel analyzed (median = 1). The lengths of plaques were highly variable; a minimum of 20 mm and a maximum of 150 mm in length were measured (median = 40 mm). In 62.5% of cases (10/16), the SFA was occluded in at least one segment; therefore, the minimum diameter was considered 0 mm. In the remaining cases, the minimum diameter registered in the most stenotic region was 1 mm in 4 cases and 2 mm in 2 cases.

### 3.2. 3D Model

The 3D-printed model (3 fixed parts and transparent lid, 1 cup for guidewire insertion) and the related gadgets (27 spacers and 3 for each configuration) required 21 h of fabrication time and 352 mL of resin. The cost of the aforementioned parts was under 200€, not including the 3D printer and curing machine writing off, energy supply, disposable materials for printing (build platform and resin tank), and post-processing (alcohol), which should have cost about 200€ related to the M-SET.

The device has never incurred damage, and there was no need to print additional components. Therefore, the average cost for the experimentation was estimated to be 400€.

**Figure 2:**
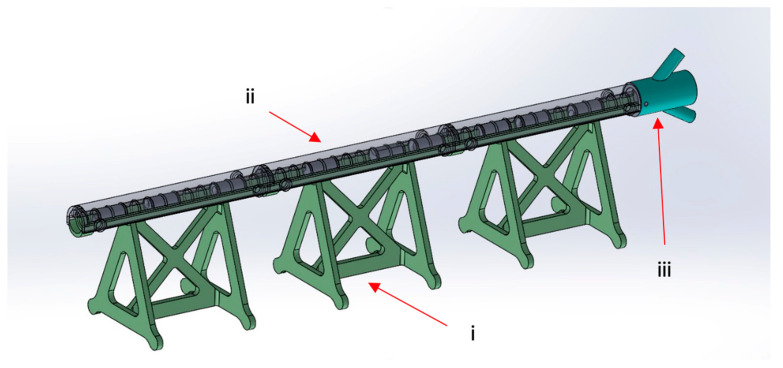
M-SET: (**i**) fixed part; (**ii**) transparent lid; (**iii**) guidewire insertion.

**Figure 3:**
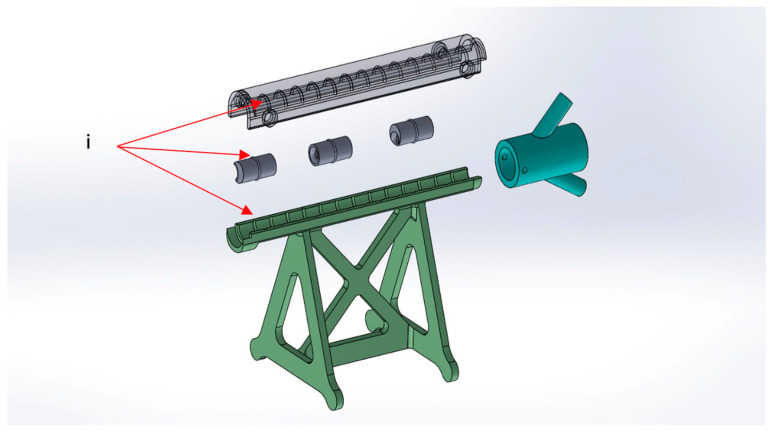
M-SET detail: (**i**) coupling system.

**Figure 4:**
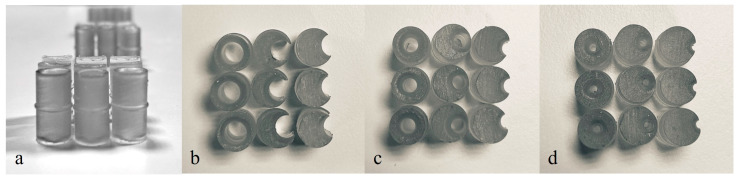
(**a**) Front view of spacers—2 cm of length. Spacer configurations (top view): (**b**) 6 mm diameter channels, (**c**) 4 mm diameter channels, and (**d**) 2 mm diameter channels.

The M-SET (Figure 2 and Figure 3), based upon the abovementioned measurements, is composed of two parts to simulate the key components of peripheral endovascular revascularization procedures:

- The vessel: 1 cm diameter tube of 14 cm in length (for each module), consisting of a fixed part that is attached to pedestals and a transparent lid that can be detached to allow spacer insertion; any module can be connected to the others, increasing the total length. At the proximal end of the model, there is a cup for guidewire insertion to simulate the introducer sheath with two tunnels of different angulations (30 and 45 degrees);
- The atheromatous plaques are cylinders with a length of 2 cm and an external diameter of 1 cm, called spacers. Each spacer is crossed by one of three different diameter channels (6, 4, and 2 mm) positioned with the circle barycenter uniformly distributed: aligned with the spacer barycenter, half radius as a distance from the center, and tangent to the perimeter. The combination of different diameters and positions of the channels allow mimicking different degrees and sites of stenosis. Due to their circular shape and coupling system, spacers can be easily turned and placed at different distances. The spacers were developed with 3 configurations (3 different positions of the channel) for the 3 different diameters of the channel (Figure 4). Spacers and channels can be customized (gate shape, tortuosity, and number of channels in the same spacer).

**Figure 5:**
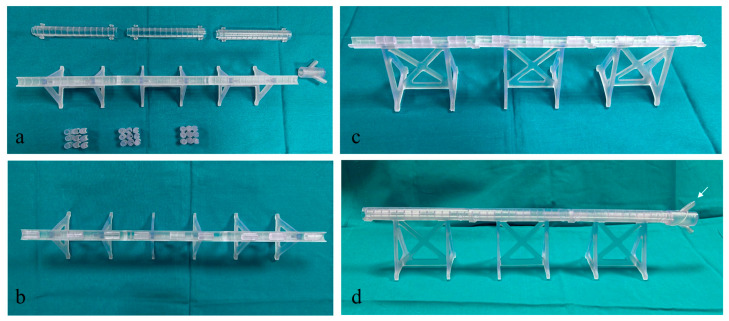
3D Model assembling stages. (**a**) 3D model components (lids, pedestals, and spacers); (**b**,**c**) different views of partially assembled model with spacers inserted in the pedestal; (**d**) a completely assembled model with a white arrow pointing at the guidewire insertion location.

These configurations allow the model to be easily assembled, as displayed in Figure 5, and to have multiple possibilities and many degrees of difficulty in the exercise (i.e., degree and number of stenosis, different orientations, and distance between spacers).

M-SET can contain up to 9 spacers in total, with 1 cm distance between each spacer. No space allowing guidewire passage between the spacers and the vessel wall was present.

For the purpose of the study, the training sessions were carried out with the M-SET assembled in the following modalities:

- Simple configuration: 9 spacers with 6 mm channels, 3 for each different channel configuration, with 1 cm distance between each spacer;
- Medium configuration: 9 spacers with 4 mm channels, 3 for each different channel configuration, with 1 cm distance between each spacer;
- Difficult configuration: 9 spacers with 2 mm channels, 3 for each different channel configuration, with 1 cm distance between each spacer;
- Random configuration: 3 spacers with 6 mm channels, 3 spacers with 4 mm channels, and 3 spacers with 2 mm channels, 1 for each different channel configuration, randomly placed inside the model, with 1 cm distance between each spacer.

Complete occlusions were not replicated because the objective was to develop spacers with directable, small channels that would allow the simulation of the endoluminal guidewire passage.

### 3.3. Evaluation and Testing

All trainees completed the task and the final simulation in a mean time of 16.24 ± 8.05 min. The mean times for the training sessions are reported in Table 2.

**Figure 6:**
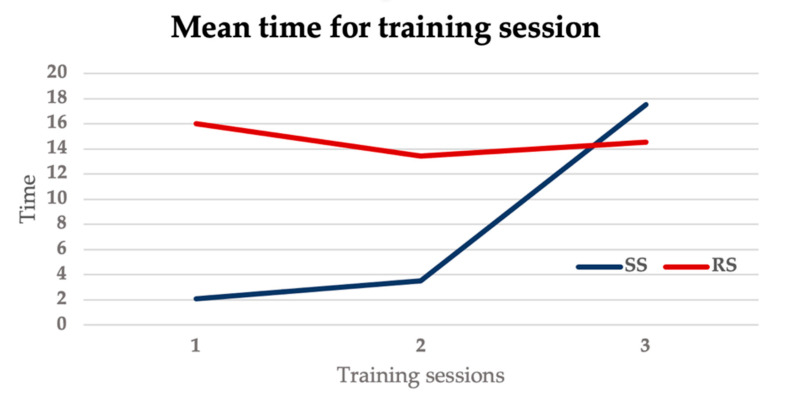
Graph showing mean time for training sessions in the SS group and the RS group.

The mean time for all the training sessions, including the non-evaluated ones, was lower in the SS group compared to the RS group (SS group: 23.13 ± 9.2 min, RS group: 44.6 ± 12.8 min, *p* = 0.0075). The mean times for the SS group in the 3 simulations show a progressive increase, whereas the times for the RS group remain stable across the three simulations, reflecting the increasing trend in the difficulty levels in the SS groups, as showed in Figure 6.

**Figure 7:**
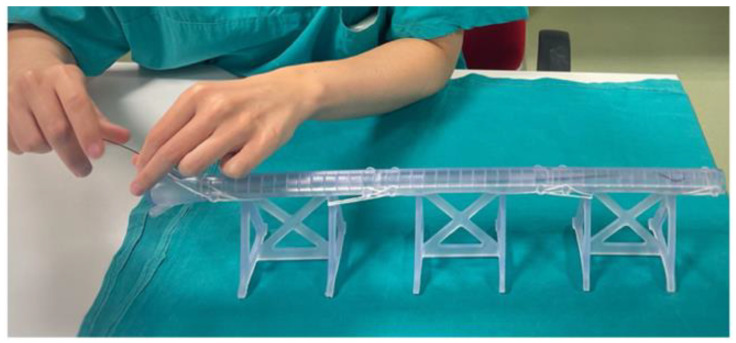
Residents performing individual training sessions. Residents granted informed consent for the pictures’ publication.

The times of the training evaluated by the senior surgeon were comparable between the two groups (*p* = 0.55). Trainees decided to use a floppy 0.035 guidewire in all cases to start the simulation, in 3/12 cases the guidewire was changed with a 0.018 or a 0.014 guidewire. A catheter was used by 4/12 trainees both to try to steer the guidewire to cross a plaque and to perform guidewires changes. The use of a torque was preferred by all trainees. Figure 7 shows trainee performing individual training sessions.

Concerning the evaluation by the Global Rating Scale of Endovascular Performance, all trainees obtained scores above the passing threshold (18 points), and the scores were homogeneous between the two groups (*p* = 1). Mean values for the Global Rating Scale of Endovascular Performance are shown in Table 3, while Table 4 summarizes responses to the post-course Student Satisfaction and Self-Confidence in Learning Scale. Both trainees’ groups were highly satisfied with the learning modules, demonstrating the usefulness of this training device and its essential role in a residency program.

## 4. Discussion

Simulation-based training has been proven to yield positive effects on learning in various medical and surgical disciplines. Specifically in surgery, simulation has demonstrated its efficacy in enhancing trainees’ performance, both in bedside practice and within the OR [57,58,59]. Simulators for endovascular intervention have become more widely available in the last few years, and the technology is evolving rapidly. However, current simulators are expensive, so their access is still limited [60,61]. Moreover, current available simulators for endovascular surgery do not focus on the development of tactile perception, which varies among individuals. 3D printing is an evolving technology that presents the advantage of producing completely customizable models while maintaining relatively low production costs.

The 3D-printed modular model for endovascular skill training presented in this study addresses this growing need for effective and affordable simulation-based training in surgery [1,21]. The model aimed to develop tactile sensations that are crucial for endovascular procedures, including torquability, trackability, and tactile feedback from the guidewire. These features are essential for replicating real-world procedural challenges.

The conceptualization and development of this 3D-printed model rest on the accurate representation of anatomical features derived from real-world patient data. The utilization of CTA from patients who underwent endovascular recanalization ensures that the model closely mimics the complexities of atherosclerotic femoral-popliteal lesions that undergo EVT. The inclusion of detailed anatomical measurements contributes to the model’s fidelity to clinical scenarios.

The modular design of the 3D-printed model and its simplicity of use allow trainees to independently perform simulations, promoting accessibility and reducing the need for additional personnel. Modularity offers a more flexible and adaptable learning experience, allowing a varying level of difficulty and customizing training sessions based on the specific needs of trainees, thereby enhancing the overall effectiveness of the simulation model. Moreover, the modularity (and the lightness of the materials) also allows for easy transportability of the M-SET. The transparency achieved through polishing the model facilitates visualization of the guidewire path through the stenosis, eliminating the need for X-rays, which ensures that this simulation model is at no risk and freely accessible at any time without the need for additional materials.

The model’s evaluation demonstrated that the trainees in the SS group, having followed a training program with progressively challenging levels, achieved results comparable to those of the RS group, despite never having executed the exercise with spacers of the highest difficulty, in nearly half of the time. This result confirms that the M-SET is a useful and effective exercise tool, particularly when performed in the comprehensive training mode, conducting simulations with increasing difficulty levels. It serves to optimize time efficiency in both training and the execution of the technical gesture.

The participants’ feedback and subjective evaluations of the 3D-printed model were generally positive. The specific simulator system used in this study was thought to be realistic, useful, and relevant. The M-SET was perceived as an encouraging factor in facilitating the learning process and was considered useful for mastering the surgical curriculum. Trainees demonstrated a high level of confidence in acquiring the necessary skills and knowledge that are essential to performing tasks in a clinical setting. The trainees also reported that the 3D-printed model was helpful in improving their understanding of the surgical technique and in enhancing their spatial perception and hand-eye coordination in guidewire handling. This finding supports the potential of 3D-printed models as an effective educational and training tool, as it is in accordance with previous experiences in the literature regarding the use of 3D-printing models in other surgical branches [62,63]. The possibility of integrating a similar training program into specialization paths and ensuring widespread use of the simulation model to enhance medical training should be considered.

### Limitations and Future Prospective

The main limitation of this study is the small size of the trainee sample involved. However, the results of the evaluation by the Global Rating Scale of Endovascular Performance, as well as the post-course Student Satisfaction and Self-Confidence in Learning Scale, were similar in the two groups, indicating the sample was homogeneous. A potential bias might arise from the fact that all trainees included in the study were from the same teaching hospital and received partial training from the authors. Multicenter randomized prospective studies are needed to increase the number of trainees and confirm the effectiveness of the proposed model on a broader scale.

Another limitation is the absence of X-ray utilization. However, the material’s “non-perfect” transparency mimics the “two-dimensional” perception of the guidewire’s path as seen on screen during EVT. However, it should be noted that the model was primarily designed to train the sensitivity of a guidewire rather than provide a comprehensive view of vascular structures.

Future research may consider the development of additional modular systems that can be integrated with M-SET to enhance realism and provide more varied scenarios, especially those involving vascular bifurcations. Multimaterial printers may simulate different plaque consistencies and surface irregularities. This would further evolve the model’s ability to replicate diverse pathological conditions.

## 5. Conclusions

Therapeutic continuity requires approaches able to assure patient centricity, continuous improvement, and sustainability. Starting from the typical healthcare focus on big data analyses and personalized medicine, progressive integration of self-safety processes, such as pre-surgical training, is required to ensure error reduction and performance improvement. In our study, bioprinting was demonstrated to be a useful approach for the development and utilization of a 3D-printed modular model for endovascular skill training. The modular setup allows training with increasing levels of difficulty and complexity. The M-SET permits the training of precise skills, ensuring their repeatability. Moreover, the validation and training program for the model, assessed by vascular surgeons and interventional radiologists, may reduce the time required to improve cognitive perception. Therefore, before customization of 3D-printed anatomical replicas, based on patient-specific data and radiological images, we suggest a tailored training to enhance precision in surgical procedures via a linear model: first, understand your perception of the path, then understand the path by using your perception.

## Figures and Tables

**Table 1:** Summary of trainees’ demographic and previous peripheral arterial revascularization experience.

Year of Residency	Age (y ± SD)	*n* = 12	n. of Previous Arterial Peripheral Revascularization Experience		
			1st Operator	2nd Operator	3rd Operator
1st year	26.5 ± 0.7	2	0	0	3
2nd year	27	1	0	2	5
3rd year	29.8 ± 1.7	6	3	10	15
4th year	29	1	4	15	20
5th year	31.5 ± 0.7	2	5	20	25
Male/Female		4/8			

**Table 2:** Mean time for training sessions in SS group and RS group.

Randomization Group	Training Sessions (Time, min.s ± SD)			
	1	2	3 (Evaluated)	Total
SS group	2.10 ± 1.74	3.49 ± 2.59	17.54 ± 8.43	23.13 ± 9.2
RS group	15.59 ± 18.54	13.42 ± 9.54	14.55 ± 8.4	44.6 ± 12.8

**Table 3:** Mean values for the Global Rating Scale of Endovascular Performance. Scale: 0 fail; 1 success, not very good; 2 success, good; 3 success, very good; 4 success, excellent, SS: standard simulation, RS: random simulation group.

Global Rating Scale of Endovascular Performance	SS Group	RS Group
Time and motion	2.33	2.66
Wire and catheter handling	2.5	2.66
Awareness of wire position	2	2.66
Maintenance of wire stability	2.66	2.33
Precision of wire/catheter technique	2.17	2
Flow of operation	2.33	2.33
Ability to complete the simulation	3.33	3.66
Need for verbal prompts	2.33	1.83
Attending takeover	1.83	1.33
Total score	21.5	21.5

**Table 4:** Summaries of the Student Satisfaction and Self Confidence in Learning Scale responses. SD = STRONGLY DISAGREE with the statement, D = DISAGREE with the statement, UN = UNDECIDED—you neither agree nor disagree with the statement, A = AGREE with the statement, SA = STRONGLY AGREE with the statement.

Satisfaction with Current Learning	SD	D	UN	A	SA
	*n* = 12 (%)				
The teaching methods used in this simulation were helpful and effective				6 (50)	6 (50)
The simulation provided me with a variety of learning materials and activities to promote my learning the medical surgical curriculum			2 (16.6)	5 (41.7)	5 (41.7)
I enjoyed how my instructor taught the simulation				4 (33.3)	8 (66.7)
The teaching materials used in this simulation were motivating and helped me to learn			1 (8.4)	4 (33.3)	7 (58.3)
The way my instructor taught the simulation was suitable to the way I learn			1 (8.4)	1 (33.3)	7 (58.3)
**Self Confidence in Learning**	**SD**	**D**	**UN**	**A**	**SA**
	***n* = 12 (%)**				
I am confident that I am mastering the content of the simulation activity that my instructors presented to me				8 (66.7)	4 (33.3)
I am confident that this simulation covered critical content necessary for the mastery of medical surgical curriculum			4 (33.3)	4 (33.3)	4 (33.3)
I am confident that I am developing the skills and obtaining the required knowledge from this simulation to perform necessary tasks in a clinical setting			2 (16.6)	5 (41.7)	5 (41.7)
My instructors used helpful resources to teach the simulation				5 (41.7)	7 (58.3)
It is my responsibility as the student to learn what I need to know from this simulation activity			1 (8.4)	3 (24.9)	8 (66.7)
I know how to get help when I do not understand the concepts covered in the simulation			1 (8.4)	5 (41.7)	6 (50)
I know how to use simulation activities to learn critical aspects of these skills				8 (66.7)	4 (33.3)
It is the instructor’s responsibility to tell me what I need to learn of the simulation activity content during class time			1 (8.4)	5 (41.7)	6 (50)

## Data Availability

The data presented in this study are available on request from the corresponding author due to privacy restrictions.
